# Notch3 in Development, Health and Disease

**DOI:** 10.3390/biom10030485

**Published:** 2020-03-23

**Authors:** Samira Hosseini-Alghaderi, Martin Baron

**Affiliations:** School of Biological Sciences, Faculty of Biology, Medicine and Health, University of Manchester, Michael Smith Building, Oxford Rd. Manchester M13 9PY, UK; samira.hosseini@postgrad.manchester.ac.uk

**Keywords:** Notch3, development, signalling, cancer, CADASIL, arteries, smooth muscle cells

## Abstract

Notch3 is one of four mammalian Notch proteins, which act as signalling receptors to control cell fate in many developmental and adult tissue contexts. Notch signalling continues to be important in the adult organism for tissue maintenance and renewal and mis-regulation of Notch is involved in many diseases. Genetic studies have shown that *Notch3* gene knockouts are viable and have limited developmental defects, focussed mostly on defects in the arterial smooth muscle cell lineage. Additional studies have revealed overlapping roles for Notch3 with other Notch proteins, which widen the range of developmental functions. In the adult, Notch3, in collaboration with other Notch proteins, is involved in stem cell regulation in different tissues in stem cell regulation in different tissues, and it also controls the plasticity of the vascular smooth muscle phenotype involved in arterial vessel remodelling. Overexpression, gene amplification and mis-activation of Notch3 are associated with different cancers, in particular triple negative breast cancer and ovarian cancer. Mutations of Notch3 are associated with a dominantly inherited disease CADASIL (cerebral autosomal-dominant arteriopathy with subcortical infarcts and leukoencephalopathy), and there is further evidence linking Notch3 misregulation to hypertensive disease. Here we discuss the distinctive roles of Notch3 in development, health and disease, different views as to the underlying mechanisms of its activation and misregulation in different contexts and potential for therapeutic intervention.

## 1. Introduction 

**Figure 1:**
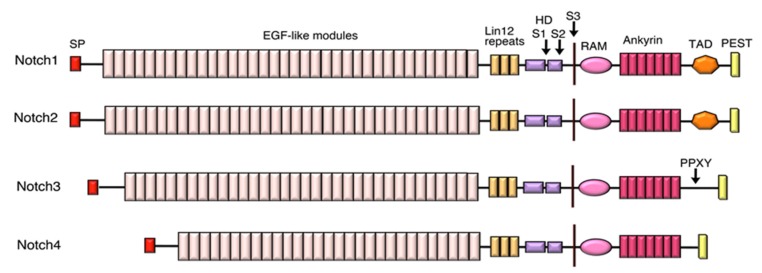
Domain structure of human Notch proteins. Schematic view comparing the domain structure of human Notch proteins. SP (signal peptide), EGF (epidermal growth factor), HD (heterodimer region), TAD (transcription factor activation domain), PEST (domain rich in proline, glutamate, serine and threonine). Locations of S1, S2 and S3 cleavage sites are indicated. PPXY motif in Notch3 acts as a WW domain recognition site for the endocytic regulator WWP2.

Notch is a transmembrane, developmental signalling receptor, which plays many crucial roles in developmental patterning, cell fate decisions, regulation of cell survival and proliferation [1,2,3]. As well as pleiotropic developmental roles, Notch receptors continue to play important roles in adult tissue maintenance and repair through regulation of stem cells and their lineages and other cellular processes. Notch was first identified in *Drosophila*, which has a single Notch gene [4]. In humans however gene duplication and diversification during evolution have given rise to four Notch orthologues (Notch1-4), which have both overlapping and specialised functions [5,6]. Figure 1 summarises the domain structure of Notch proteins, which is highly modular in nature. The large extracellular domain (ECD) is comprised largely of tandem repeats that are closely related to epidermal growth factor (EGF) and vary in number between different Notch homologues. These EGF-modules are characterised by 6 highly conserved Cysteines, which form three stereotypical disulphide bonds, contributing stability to the EGF-module fold. Notch1 and Notch2 each possess 36 EGF modules similar to *Drosophila* Notch. Notch 3 and 4, however, have smaller extracellular domains (ECDs) with 34 and 29 respectively. Further differences are located in the intracellular domain (ICD) which is shorter for Notch3 and 4 and lacks a region thought to act as a transcriptional activator domain (TAD) (Figure 1). More proximal to the membrane, there is a negative regulatory region (NRR) whose domain structure buries a proteolytic site [7] and this plays a key role in the activation mechanism of Notch.

**Figure 2:**
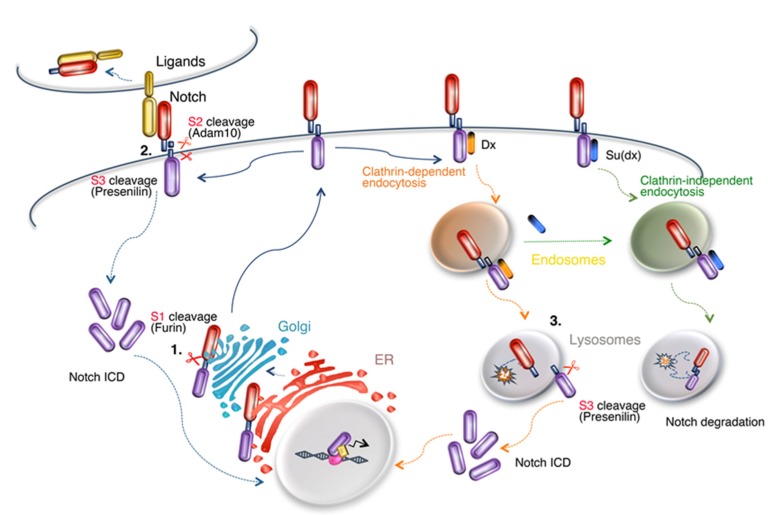
**The life cycle of Notch.** Schematic model of the synthesis, secretory and endocytic transport, activation and turnover of Notch. Locations in the cell where proteolytic cleavages can occur are indicated (**1–3**) and may be relevant to detachment of the Notch3 extracellular domain (ECD). (**1**) Notch is cleaved by furin while in transit in the Golgi to form a processed heterodimer. (**2**) At the cell surface, ligand interaction promotes exposure of the S2 cleavage site, which is a substrate for Adam10-dependent cleavage. The released ECD is endocytosed, along with bound ligand, into the signal-sending cell. (**3**) In *Drosophila*, Notch endocytosis is by both clathrin-dependent and -independent routes [20] respectively promoted by ubiquitin ligase regulators deltex (Dx) and suppressor of deltex (Su(dx)). Dx promotes ligand-independent activation of Notch following removal of ECD by an Adam10-independent mechanism and presenilin-dependent release of intracellular domain (ICD) through S3 cleavage. Su(dx) promotes Notch transfer to intraluminal vesicles of the endosome and degradation of the full-length receptor on lysosome fusion.

Proteolytic cleavage is an essential feature of the mechanisms by which Notch is activated (Figure 2). Notch proteins are normally first cleaved by furin in the S1 site of the extracellular domain during secretory transport, leaving the full-length receptor to reach the cell membrane as a processed heterodimer, although the requirement for this process may vary between different Notch proteins [8]. In the canonical signal activation mechanism Notch is then activated at the cell surface by binding of membrane ligands of the Delta or Serrate/Jagged families defined by a common Delta/Serrate/Lag2 (DSL) domain. Ligand endocytosis-induced tension on the tightly bound Notch extracellular domain (ECD) results in local unfolding at the NRR and thus exposure of the S2 cleavage site [9,10]. The latter is recognised and cleaved by Adam10 metalloprotease, with the ECD being removed and transendocytosed into the signal-sending cell still bound with the ligand (Figure 2). The remaining membrane tethered intracellular domain with its small extracellular moiety then becomes a substrate for the gamma-secretase complex which acts to cleave Notch at the S3 site in its membrane spanning sequence [11]. This releases Notch intracellular domain (ICD) to translocate to the nucleus where it forms a complex with the transcription factor CBF-1/Suppressor of Hairless/Lag1 (CSL), along with additional cofactors that activate gene transcription (Figure 2). Additional routes to activation have also been described in both invertebrate and vertebrate models, which are independent of ligand binding [12,13,14,15,16,17,18]. These ligand-independent mechanisms, best characterised in *Drosophila*, depend on endocytosis of full-length Notch and proteolytic activation in the endosomal and lysosomal membranes (Figure 2) [13,14,19,20].

The evidence to date indicates that Notch3 is activated by DSL domain-containing stereotypical ligands according to the above described mechanism [21,22,23,24]. Recent reports indicate that Notch3 also has a high background of ligand-independent signalling which may be relevant both to normal Notch3 functions and in pathological contexts [25]. The mechanism is unknown, but the native NRR fold of EGF was reported to be less stable than that of other Notch proteins studied. Whether this ligand-independent mechanism depends on Notch3 endocytosis similar to Drosophila Notch activation mechanisms is not known. It is also not ruled out that non-canonical ligands might be involved. For example, YB-1 is a cold shock domain-related protein, which is reported to activate Notch3 through binding to EGF-modules 20–23 and may regulate immune and inflammatory responses [26,27].

## 2. Developmental Roles of Notch3

**Figure 3:**
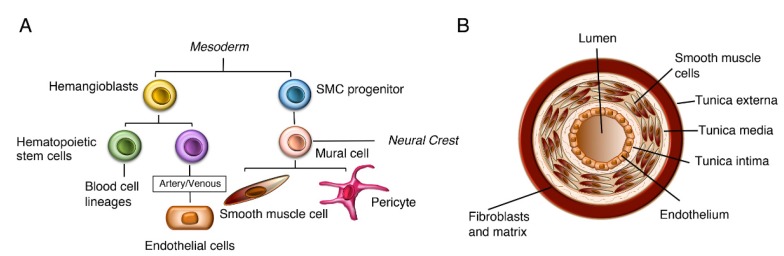
Lineage and role of vascular smooth muscle cells. (**A**) Lineage of vascular smooth muscle cells (VSMCs) showing relationship between different cell types that comprise the blood vessels. VSMCs are derived from both mesodermal and neural crest origins. (**B**) Schematic view of cross-section of an artery indicating layered structure of the vessel wall.

Early genetic studies on mice lacking Notch3 showed them to be viable and fertile with no reported phenotypes [28]. However, later studies indicated developmental roles for Notch3 in the vasculature and in particular the lineage leading to vascular smooth muscle cell fate. During vascular development, endothelial precursor cells differentiate and form tubular networks. During angiogenesis, the networks undergo sprouting, and vessels are stabilised by recruitment of mural cells, which differentiate into vascular smooth muscle cells (VSMCs) (Figure 3) [29]. A detailed analysis of post-natal Notch3 null mice found that there were defects in smooth muscle cell maturation, arterial differentiation and morphology compared to wild type mice leading to thinner and improperly formed smooth muscle cell layers that normally surround the arterial vessels [30]. Published work from in vitro studies indicates that Jagged1 expression in endothelial cells activates Notch3 in VSMCs with an autoregulatory loop maintaining Notch3 expression in VSMCs [23,24]. A key downstream effector in this process is the PDGF (platelet derived growth factor receptor) signalling pathway and PDGFR- is upregulated by Notch3 activation [31]. Further work has shown that Notch3 also has roles at earlier steps in the VSMC lineage, which are masked by partial redundancy with other Notch homologues. In zebrafish, Notch2 and Notch3 act together to regulate production during embryogenesis of both mesoderm-derived and neural crest-derived mural cells, the precursors of VSMCs [32]. A similar redundancy is found in mice as Notch2, Notch3 double mutants are embryo lethal with severe loss of VSMCs and vascular abnormalities [33]. However further work using primary cell cultures indicated some distinct roles of Notch2 and Notch3 within the VSMCs. Notch2 suppresses VSMC proliferation, while Notch3 promotes proliferation and protects against VSMC apoptosis [34,35]. Functional overlap and interrelationship between different Notch proteins are thus complex and highly context-dependent.

A combination of heterozygous Notch1 mutation with Notch3 deficiency in mice has also revealed a requirement for Notch3 in forming pericytes, an alternative differentiated mural cell fate [36]. Pericytes are recruited to the vasculature during angiogenesis and contribute to basement membrane formation, which stabilises the vessels. The loss of Notch3 enhanced hypervascularisation phenotypes in the mouse retina that occur with heterozygous Notch1 mutants and caused disorganisation of the basement membrane and arteriovenous malformations, with inappropriate direct connections between arterial and venous systems [36]. In zebrafish, the deletion of Notch3 alone led to more severe effects than in mice, with a loss of pericytes, haemorrhage, and defective blood–brain barrier. The likely role for Notch3 in this case was proposed to be pericyte proliferation during embryogenesis rather than initial specification [37]. Notch3 was also found to have a role in mouse cardiac smooth muscle development but in this case the details of the lineage differ, as the smooth muscle cells (SMCs) are derived from pericytes, which initially coat the cardiac artery vessel walls, having migrated from the epicardium [38]. In mice deficient for Notch3, pericardium recruitment occurs normally but their differentiation into cardiac smooth cells is impaired. This normally occurs at sites of arterial remodelling on initiation of blood flow in response to endothelial expressed Jagged1, whose expression is also upregulated on initiation of blood flow [38]. A similar pericyte conversion may also occur during kidney development [38]. In zebrafish an additional redundant development role of Notch3 has been uncovered by examination of phenotypic consequences of knockdown of Notch3 expression in Notch1a mutant fish embryos. This revealed a contribution of Notch3 to the development of rhombomere boundaries within the hindbrain regions and additional neuronal hyperplasia [39].

## 3. Roles of Notch3 in the Adult Organism

As with other Notch proteins, Notch3 continues to function in various capacities in the adult organism, further highlighting functional interactions, cooperation and specialisations of different Notch proteins. Notch3-deficient adult mice show further progressive phenotypes in brain and retinal vasculature, resulting from vascular smooth muscle cell degeneration and loss through apoptosis [40]. This causes loss of vessel integrity, haemorrhage and loss of blood–brain barrier function. *Notch3* expression in mouse vascular smooth muscle cells also plays a role in regulating vascular tone and flow-mediated dilation on cerebral and tail resistance arteries [40]. Furthermore, *Notch3* deficiency alters the physiological adaptation to high blood pressure in adult mice. *Notch3*-deficient mice were found more likely to develop heart failure when maintained in a hypertensive condition [40]. This was found to reflect reduced F-actin content and a decline in contractile phenotype of the VSMCs after systemic hypertension was induced with angiotensin II treatment. A reduced capillary density was observed and heart failure was associated with increased oxidative stress.

These findings support a role of Notch3 in mediating mechanotransduction. Phenotypic plasticity of VSMCs in response to changes in haemodynamic environment is important in healthy vascular function. When VSMCs are in a contractile condition they regulate vascular tone, but in adaption to mechanical inputs VSMCs can switch morphology to a synthetic phenotype and contribute to vascular growth and remodelling [41]. An interesting recent study combining experimental observations and computational modelling has revealed that Jagged-1-induced Notch3 signalling decreases with increased mechanical load with a switch between contractile and synthetic VSMC phenotype linked to changes in thickness in the VSMC wall that encloses the endothelial cells that form the artery vessel (Figure 3) [42]. Further in vitro studies have linked shear stress to the signalling potential of endothelial cells through regulation of Jagged-1 endocytic trafficking [43].

Adult roles of Notch3 extend beyond the vasculature. Recent work has identified the function of Notch3 in neuronal stem cells and neuronal differentiation [44,45]. The subependymal zone is an important stem cell niche in the adult mammalian brain, containing active and quiescent stem cell populations. There is a division of labour between Notch1 and Notch3 in regulating these populations. In studies of the mouse model system, Notch1 is preferentially expressed in active stem cells and promotes their proliferation, while Notch3 is preferentially expressed in quiescent stem cells and is required for their maintenance by suppressing their proliferation [42]. Similar outcomes and relative roles for Notch1 and Notch3 in neural stem cell regulation were identified in the zebrafish model [43].

Notch3 also plays a role in the regulation of satellite cells, which are stem cells involved in skeletal muscle repair. Notch3 null mice showed considerably more muscle growth than wild-type mice after repeated injury associated with increased proliferation of activated satellite cells [46], whereas Notch1 has been positively linked with satellite cell activation and proliferation [47]. Similarly, in the mouse mammary gland, Notch3 has been linked to restricting the proliferation of a luminal population of stem cells [48]. Notch3 also has roles in oesophageal homeostasis in cooperation with Notch1. Notch1 signalling activates Notch3 expression, which in turn promotes oesophageal squamous cell differentiation [49].

## 4. Notch3 and Disease

### 4.1. Notch3 and Cancer

Aberrant Notch signalling was first linked to human cancer through the identification of a chromosome translocation, in T-cell acute lymphoblastic leukaemia (T-ALL), that resulted in the expression of the soluble cytoplasmic domain of Notch1, which was constitutively active, linking ectopic Notch activity to cancer progression [50]. While this type of mutation has, however, proven to be quite rare, it has subsequently been found that about 50% of T-ALL harbour activating mutations in Notch1 [51]. These are largely comprised of missense mutations in the NRR domain of Notch1 or C-terminal truncations that remove the PEST domain which is involved in Notch protein turnover. Notch3 has also been implicated in T-ALL, and NRR and PEST (proline, glutamate, serine, threonine-rich) domain mutations have been detected in T-ALL cell lines [52]. However, only a few mutations have been identified from patient samples. Instead, overexpression of Notch3 is associated with the disease in nearly all cases [53,54]. Blockade by antibodies specifically targeting the Notch3 NRR domain has been shown to have anti-leukemic effects [52]. Notch1 and Notch3 signalling has been found to invoke a similar downstream oncogenic programme including the driving of the cell growth regulator Myc [55].

A number of different factors are reported to drive high Notch3 expression in T-ALL. Activated Notch1 signalling has been shown to drive Notch3 expression, but high Notch3 is also present in T-ALL without activating Notch1 mutations [56,57]. Recent work has implicated Notch3 acting in a self-sustaining feedback loop to maintain its high expression combined with reduced histone3 (H3) K27 methylation, increased H3K4 methylation and K27 acetylation of the Notch3 enhancer region, an activated gene expression profile [56,57]. This is in part regulated by aberrant expression of the epigenetic regulator Boris (brother of regulator of imprinted sites) [58]. Post-transcriptional regulation by micro-RNAs is also important for the regulation of Notch3 expression, for example by miR-150 [59]. In T-all cells miR-150, normally highly expressed in the lymphoid lineage, is reduced in expression and this loss may therefore contribute to increased Notch3 levels [60] as has also been found in lung adenocarcinona and ovarian cancer [61,62].

Aberrant Notch3 protein levels in T-All are also associated with altered protein turnover. Histone deactylase 6 (HDAC6) has been shown to have a number of alternative substrates apart from any chromatin modification role [63]. Recent work has indicated that HDAC6 promotes high levels of Notch3 expression and blocking the activity of HDAC6 increased lysosomal degradation of Notch3. This may be through interference with tubulin deacetylation, a known substrate for HDAC6 [57]. Other work has shown that Notch3 itself is acetylated and deacetylated by p300 and HDAC1, respectively, to regulate the proteosome-dependent degradation of Notch3 ICD, and inhibition of HDAC1 has anti-leukaemic properties [64].

Unlike Notch1, which is mutated to become ligand-independent, Notch3 is rarely mutated in T-ALL [52]. Like other Notch proteins, Notch3 signalling is stimulated by binding of ligands of Delta and Serrate/Jagged class of membrane bound proteins, which share the Delta/Serrate/Lag2 (DSL) domain. Notch3 also displays significant basal levels of signalling activity, independently of ligand interactions [25], and it is possible that both modes of activities play a role in T-ALL. It has been suggested that Delta-like 4 (Dll4) expressed on endothelial cells may play an activating role in promoting T-ALL in a tumour model system in mice that revealed the roles of Dll4 in mediating T-ALL release from tumour dormancy [65]. Dll4 expression in the bone marrow may be another source of this ligand, which may come into contact with Notch1 or Notch3 in T-ALL cells [66]. Another study points to a potential role for cis-expressed Jagged1, i.e., expressed in the same cells as Notch3. Normally cis-expressed ligands are associated with Notch inhibition rather than activation, but in this context, it is reported that cis-Jagged1 expression promotes Notch activity by two mechanisms [67]. Firstly, the Jagged1 intracellular domain was found to be cleaved from a lipid raft membrane compartment by Adam10 to relocate to the nucleus and to participate in Notch3 target gene regulation through binding to the CSL transcription factor, along with Notch3 ICD. Secondly, the Jagged1 extracellular domain was found to be released into the extracellular medium and activate Notch3 in surrounding cells. It is not clear how soluble ligand overcomes the normal requirement for ligand endocytosis to cause NRR unfolding, but perhaps the reduced stability of the NRR of Notch3 has a role here. A self-sustaining positive feedback loop was proposed because Notch3 signalling had been found to drive Jagged1 expression [67].

The ability of Notch3 to activate in the absence of DSL ligands suggests that it is likely that this constitutive activation mechanism plays a role in Notch3 signalling in cancer, particularly, given the high expression levels of Notch3 that are often observed. Ligand-independent activation of Notch3 in T-ALL has not been specifically investigated to our knowledge, but it has been reported to be significant in breast cancer [68], which, like T-ALL, is frequently associated with high Notch3 expression. Breast tumours with high Notch3 levels are enriched in the triple-negative breast cancer (TNBC) subclass that lacks estrogen receptor, progesterone receptor (PR) and human epidermal growth factor receptor 2 (Her2) expression. About a third of TNBC is associated with Notch3 gene amplification/overexpression and overactivated Notch3 signalling, and Notch3 seems to be the primary Notch signal associated with TNBC [68,69,70]. C-terminal truncations of Notch3, associated with increased stability of the Notch3 ICD, have also been identified in tumours with high Notch3 expression [70]. Similarly, Notch3 gene amplification and expression are associated with ovarian cancer and Notch3 signalling has been shown to promote breast and ovarian tumour cell growth, survival and metastasis [68,69,71]. Interestingly, reduced Notch3 endocytic turnover has also been shown to play a role in stimulating Notch3 activity in ovarian cancer cells. Mutations removing the Nedd4 family ubiquitin ligase gene WWP2 are commonly found in ovarian cancer and lost WWP2 activity is associated with decreased Notch3 lysosomal degradation and increased signalling activity [71]. WWP2 binds to Notch3 via a PPXY motif (Figure 1) that recruits WWP2 through its WW domains. In this regard, the regulation of Notch3 appears similar to *Drosophila* Notch, whose endocytic-dependent degradation is dependent on Suppressor of deltex (Figure 2), a *Drosophila* Nedd4 family protein, which regulates a ligand-independent activation mechanism [12,20].

It should be noted, however, that contrary reports of the role of Notch3 in breast cancer have also been published. For example, in estrogen receptor-α positive tumour cells, Notch3 activity has been reported to suppress epithelial–mesenchymal transition and reduce metastases [72]. Notch3 expression and signalling have also been linked to the promotion of cellular senescence and a tumour suppressor role of Notch3 has been proposed. The details of cellular context are likely to account for the different outcomes reported in different studies.

### 4.2. Notch3 and Cerebral Autosomal Dominant Arteriopathy with Subcortical Infarcts and Leukoencephalopathy (CADASIL)

CADASIL is an autosomal, dominantly inherited small vessel disease [73,74,75,76]. Genetic defects are located within Notch3 and are associated with accumulation of its extracellular domain (ECD), detached from its intracellular domain (ICD) in aggregates associated with VSMCs [77,78,79,80]. CADASIL is a progressive disease associated with vascular abnormalities affecting arterial VSMCs. Depositions of granular osmiophilic material (GOM), which include Notch3 ECD and other components [79], occur in the VSMC basement membrane and there is degeneration and loss of VSMCs through apoptosis or altered proliferation [81,82]. These effects are widespread in the vascular system, but the pathological consequences are focussed on the brain. Early symptoms include migraine with aura (mean onset in the twenties), progressing to transient ischaemic attacks and stroke (mean onset in forties), and ultimately symptoms of vascular dementia with white matter lesions and fluid filled cavities (lacunes) associated with deep brain infarcts [73,74,75,76]. Defects in blood–brain barrier function associated with pericyte loss have also been suggested, but this may not be a consistent feature [83].

Most CADASIL mutations either substitute or introduce a cysteine within Notch3 EGF modules, potentially destabilising the disulphide bonding pattern and module fold [74]. While CADASIL is reported to affect about 4/100000 of the population, genomic studies have revealed that about 1/300 individuals carry archetypal CADASIL mutations [84,85] and it is possible that the condition is considerably underdiagnosed or has a wider spectrum of severity than previously appreciated. Supporting the latter, the distribution of mutations in the general population differs from that of diagnosed CADASIL cases. From studies of diagnosed CADASIL cases, it has been found that there is a pronounced clustering of disease-associated mutations in the five N-terminal EGF modules, while population genomics studies show less clustering in the N-terminal region [84,85]. A comparison of clinical data suggested that CADASIL mutations affecting the N-terminal region tended to have more severe outcomes and earlier disease onset. It is not known whether there is a specialised regulatory significance of the N-terminal region of Notch with some specific relevance to disease progression. Genetic studies have shown that *Drosophila* Notch mutations that disrupt disulphide bonds of differently located EGF modules indeed have different functional outcomes, which can be a loss or gain of function for signalling with different outcomes on developmental cell fate decisions [86,87]. Notwithstanding the above reports, published work has shown that the CADASIL mutation C445R, which lies in EGF11, within the ligand-binding region of Notch3, has a more severe outcome and early-onset phenotype [88]. However, other ligand-binding region mutations were associated with less severe cognitive impairments compared to more commonly located CADASIL mutants, although paradoxically with larger volumes of white matter lesions detected by magnetic resonance imaging [89]. The contrasting observations are relevant to understanding the connection between mutations of Notch3 and the manifestation of the consequences on VSMCs, a pathway, which remains the subject of debate and alternative models in the literature.

Research work in cell culture has shown that differently located mutations have different effects on the ability of Notch3 to signal [90,91], with some CADASIL mutants apparently having no effect on signalling, but others located around the ligand-binding region blocking ligand-induced signalling. This disconnection between effects on Notch3 signalling and disease occurrence has led to a model of neomorphic toxicity, resulting from abnormal recruitment of extracellular matrix components, rather than a signalling defect [79,92]. Notch3 ECD has been reported to be deposited in disulphide cross-linked aggregates that sequester matrix components, such as tissue inhibitor of metalloproteinase 3 (TIMP3), vitronectin and latent TGF-β-binding protein 1 (LTBP-1). Support for this model comes from studies on a transgenic mouse Notch3 CADASIL mutant model, in which genetically reduced TIMP3 and vitronectin levels were able to partially ameliorate different phenotypic consequences, while not affecting Notch3 ECD deposition. A reduction in TIMP3 levels suppressed Notch3 mutant effects on myogenic tone, while a reduction in vitronectin reduced white matter lesions [93]. Altered TGF-β signalling has been found in a related, rare, recessive, small vessel disease CARASIL (cerebral autosomal recessive arteriopathy with subcortical infarcts and leukoencephalopathy), and increased TGF-β signalling has been proposed to reduce VSMC proliferation in CADASIL as a downstream consequence of Notch3 ECD deposition [82].

Other research studies have favoured a model in which a deficit in Notch signalling contributes to the CADASIL phenotype. For example, dominantly inherited truncation and nonsense mutations have been reported to be associated with small vessel disease without Notch aggregation and GOM [94,95]. However, other studies have concluded that hypomorphic Notch alleles are not associated with CADASIL features [96]. The spectrum of phenotypic variability, age of onset and penetrance of small vessel disease linked to Notch3 defects need further investigation to fully evaluate these conflicting results. Even in cases with stereotypical CADASIL mutations, the presence of Notch3 ECD aggregates in skin biopsies is not fully diagnostic with about 80%–90% sensitivity reported [97]. Interestingly, in one study, an individual was identified with clinical features similar to that of CADASIL, but who was homozygous for a null Notch3 mutation [98]. From magnetic resonance imaging (MRI) scans, there was evidence of white matter lesions and deep brain infarcts, while examination of skin and muscle blood vessels revealed changes in small vessel walls, including loss of VSMCs. No GOM deposits were observed in this case, suggesting that some features of CADASIL may not depend on protein aggregation [98]. Some studies in mouse models have also suggested that the loss of Notch3 signalling may contribute to CADASIL phenotypes [99], since Notch3 null mutant mice show a progressive loss of VSMCs, blood–brain barrier disruption and some local losses of vessel integrity. No GOM deposits were observed. However, in another study, clinical manifestations such as white matter lesions were not identified in Notch3 null mice [100], although similar white matter lesions were found in transgenic mouse models expressing a human Notch3 CADASIL mutant [100].

Interestingly, in Notch3 null mutant mice, which are also heterozygous for a Notch1 mutant, an enhanced phenotype was observed which was reported to include GOM in neonatal mice, which the authors attributed to an accelerated CADASIL phenotype [36]. This work suggests that some Notch1 activity may in part compensate for the compromised Notch3 activity in CADASIL patients. It also indicates that when combined Notch signalling is strongly compromised, then GOM deposition can occur independently of Notch3 ECD aggregates. Some previous biochemical analyses have suggested that Notch3 ECD can form dimers with other Notch proteins and have a dominant negative effect on Notch signalling generally and this may be exacerbated by CADASIL mutations, which are cleared more slowly from the cell [101]. However, different mouse studies indicated that no change or loss of Notch signalling is associated with the expression of CADASIL-relevant phenotypes [100,102].

Given the alternative views supported in different studies, it is likely that the contribution of Notch3 mutations to CADASIL phenotype is complex and multifaceted and the balance of contributions from signalling, dominant-negative effect and neormorphic toxicity may differ for different mutations. It is important to further understand this heterogeneity when considering possible therapeutic solutions. Two antibody approaches have been tested recently. Assuming that CADASIL mutant phenotypes result from reduced Notch3 signalling, one approach tested the use of an antibody to the Notch3 NRR domain, which destabilises the domain and stimulates ligand-independent signalling [103]. In a mouse model bearing the C445R CADASIL mutant that blocks ligand-induced signalling, the use of the agonist antibody recovered phenotypes, such as mural cell coverage of arteries, that result from Notch signalling deficits [103]. However, the consequences of agonist antibodies were not investigated on more archetypal, N-terminally located, CADASIL mutants that have been reported not to have Notch signalling deficits. An alternative approach targeting the proposed toxic effect of Notch3 aggregates has also been tested using an antibody against the Notch EGF module-containing region of the ECD [104]. The aggregates in the mouse transgenic CADASIL model were successfully decorated with the antibody and some reversal of impaired cerebral blood flow responses were also achieved. However, the treatment did not prevent the formation of white matter lesions. Therefore, while both of the above-mentioned models of CADASIL have support in the literature, questions still remain as to whether either model provides a complete understanding.

A recently published study further complicates the interpretation of the CADASIL phenotype and provides an alternative explanation [105]. An examination of VSMC primary cultures isolated from CADASIL patients identified increased Notch3 signalling was identified through quantification of Notch3 downstream target HEYL, which was suppressed by gamma-secretase inhibitors. CADASIL VSMCs were found to have increased cell proliferation, increased apoptosis and altered cytoskeletal morphology. Downstream effects of Notch3 activation were identified to include increased Rho kinase activation (mediated by increased ROCK2 expression), altered Ca^2+^ transients and increased endoplasmic reticulum (ER) stress. The latter was mediated by increased expression of ER stress genes, including Bip, and ER-localised chaperones. This increase was abolished by gamma-secretase inhibitors. The intermediate between ER stress and Notch3 appears to be Notch3-driven expression of NADPH oxidase 5 (Nox5), which regulates the production of reactive oxygen species (ROS) [105]. Rho kinase activity and ER stress were linked to changes in VSMC morphology but not to proliferative responses to Notch3. This human study was further supported by data showing Notch3 activation in the transgenic Notch3R169C mouse CADASIL model [105].

A similar conclusion regarding the increased activation of Notch3 in CADASIL was obtained from studies of VSMCs differentiated from induced pluripotent stem cells (iPSCs) generated from the fibroblasts of a CADASIL patient [106]. In this study, increased Notch activity was also linked to increased VSMC proliferation and cytoskeletal reorganisation. The upregulated expression of genes linked to the NF-kB pathway was identified, indicating that an inflammatory response may be involved. Another recent study using patient-derived iPSCs showed that CADASIL mutant Notch3 mural cells failed to stabilise endothelial networks (also iPSC- derived) in a co-culture model, unlike wild-type VSMCs [107]. The CADASIL VSMCs were also more prone to apoptosis. The defects were reversed by siRNA knockdown of Notch3 expression, indicating a gain-of-function effect, although in this case neomorphic or hypermorphic activity was not discriminated. The mechanism by which a gain of Notch3 signalling arises from archetypal CADASIL mutations is not yet explained. Interestingly certain Cysteine missense mutations in Drosophila Notch are linked to gain-of-function activity, and exploring further the mechanisms of this signal activation may provide a useful model system to identify the process involved [86]. The current understanding of the links between Notch3 mutations and CADASIL disease is thus represented by three alternative views which differ concerning the relationship between Notch3 signal alteration and the disease, and the alternatives are loss of function, irrelevant (neomorphic activity), and a gain of Notch3 activity. Further research is required therefore to understand the primary mechanistic defect arising from Notch3 mutations to discriminate further between these models. It is possible that a combination of consequences may be involved.

The nature of the primary defect that leads to Notch3 ECD accumulation is less intensively studied but offers an alternative route to developing a therapeutic strategy if this gap in our knowledge could be filled. Whatever the proposed model, it must take account of the predominance of the cysteine altering mutations associated with the CADASIL disease. The "life cycle of Notch" offers a number of possible points in which Notch3 ECD separation could occur (Figure 2). During trafficking to the cell-surface, Notch proteins are cleaved by Golgi-resident furin proteases at the S1 cleavage site to form a processed heterodimer. The latter is held together by calcium-dependent, non-covalent interactions [108]. The dissociation of the heterodimer components could feasibly occur during the secretory process, particularly if disulphide-bonded cross linking involving the odd number of cysteines in CADASIL mutants altered the structure and interactions of the ECD. At the cell surface Notch3 interaction with ligands induces ECD separation after S2 cleavage as part of the normal activation mechanism and ECD is then normally trans-endocytosed into the ligand-bearing cell along with the bound ligand. Alternatively, Notch3 ECD might be removed following endocytosis as part of a ligand-independent activation process akin to that found in *Drosophila* [13,20]. The defective clearance of disulphide cross-linked ECD at any of these points might be responsible for ECD accumulation and it is possible that there is more than one mechanism involved. Previous studies have suggested that CADASIL mutant Notch3 aggregates arise in the ER [109]. However, this study used an inducible overexpression system in HEK cells and wild-type Notch3 also exhibited aggregates although to a lesser degree. Only Notch3 ECD localisation was assessed by immunofluorescence and hence authentic CADASIL-like accumulations were not verified. However, a decrease in turnover of the mutant protein does suggests that, wherever Notch3 aggregates form, defective clearance of the mutant ECD may contribute to its accumulation [109]. Another study, also using Notch3 expression in HEK cells suggested that, after ligand interaction from adjacent cells, there was defective transendocytosis of Notch3 into the ligand bearing cell and an increased lifetime of Notch3 ECD at the cell surface [110]. This difference was only observed when Notch3 expressing cells were in co-culture with ligand expressing cells. In this study, Notch3 was normally transported to the cell surface as an S1 processed heterodimer. It is not clear whether accumulated ECD material remained in this form or already detached from ICD, although the latter would be expected to release it from the membrane. Despite this ligand-induced Notch3 signalling was not impaired by the CADASIL mutations [110]. However, with regard to ligand involvement in Notch3 ECD separation in CADASIL then it is noted that some CADASIL mutants are defective for canonical ligand binding [88,102] and so, while this step may contribute to ECD separation, then it appears not to be essential. Other studies have implicated the endo/lysosomal pathway in mistrafficking of CADASIL mutant Notch3. In an electron microscope study of post-mortem human brain and skin biopsy samples an accumulation of ubiquitin was noted in VSMCs and GOM was often located within membrane invaginations resembling sites of endocytic vessel formation [111]. It was suggested that defective endocytosis of CADASIL Notch3 may be involved in Notch3 aggregate formation. Other studies, using primary human tissue derived VSMC primary cell cultures, have indicated that CADASIL mutant Notch3 may accumulate intracellularly through defective autophagosome/lysosome fusion [112].

Given the uncertainties regarding mechanisms of primary defects and how the pathological outcomes arise, some groups have advocated mutation correction as a way forward for therapeutic approaches, including inducing exon skipping to remove the mutant EGF module region [113]. Intriguingly a naturally occurring exon skip was identified to remove exon 9 in a human patient with a mild form of the CADASIL disease arising from a heterozygous G498C mutation in EGF12, part of the ligand binding region. This mutation coincidentally lies adjacent to the exon 9 donor splice site. Exon skipping efficiently produced a ligand-binding defective form of the protein with only a small fraction of Notch3 produced bearing the cysteine altering mutation.

### 4.3. Notch3 and Pulmonary Hypertension

Pulmonary hypertension (PH) results from arterial vessel wall thickening and lumen occlusion in response to increased VSMC and endothelial cell proliferation. It is a progressive disease, which leads to increased pulmonary arterial pressure and death. Notch3 expression is elevated in VSMCs from lung biopsies of PH patients and rat and mouse models with experimentally induced PH [114]. Studies in mice indicated that increased expression was specific to the lungs. Notch3 signalling through the Hes5 target gene was increased and this was found to drive increased proliferation of pulmonary artery smooth muscle cells. Notch pathway inhibition reversed PH in mouse models and Notch3 null mutant mice did not develop PH [114]. Further work has indicated that Notch3 activation lies downstream of mTor pathway activation, at least in a hypoxia-induced mouse model of PH [115]. PH is also one of the consequences of extra-uterine growth restriction in early post-natal life and a recent study has linked increased Notch3 signalling to pulmonary smooth muscle cell proliferation in this disease context as well [116].

PH also has a genetic origin and inherited forms are associated with mutations in TGF-β pathway components. However, one study has linked PH to mutations in Notch3 [117]. Mutations in conserved residues of Notch3 ECD (G840E and T900P) were identified in cases of childhood PH. Interestingly, these mutations are located in a region of Notch3, which aligns with an EGF-module region of Drosophila Notch in which gain of function mutations have been identified [86], which would support the model of PH being associated with increased Notch3 activity. In this study, however, only decreased signalling was observed when luciferase Hes5-Notch reporter assays were carried out, but it remains possible that this reflects the context of the HEK cell Notch3 overexpression model cell line used.

## 5. Conclusions

Notch3 has perhaps been less intensively studied than other members of the Notch family that have more obviously pleiotropic roles. Nevertheless, the importance of Notch3 to human health is becoming increasingly clear as new developmental functions previously masked by redundancy are revealed and the contribution to adult tissue homeostasis is becoming more appreciated. As with other Notch proteins, Notch3 has many links to human diseases through mutation, altered expression or misregulation of its activity or turnover. The duplication of Notch genes and diversification of their function have likely led to specialisations of particular mechanisms of their regulation. Further studies of the ways in which Notch3 is uniquely regulated compared with other human Notch proteins will underpin the development of means to more specifically target its aberrant activity in different disease contexts.

## References

[1] Kopan R., Ilagan M.X. (2009). The canonical Notch signaling pathway: Unfolding the activation mechanism. Cell.

[2] Bray S.J. (2016). Notch signalling in context. Nat. Rev. Mol. Cell Biol..

[3] Baron M. (2017). Combining genetic and biophysical approaches to probe the structure and function relationships of the Notch receptor. Mol. Membr. Biol..

[4] Wharton K.A., Johansen K.M., Xu T., Artavanis-Tsakonas S. (1985). Nucleotide sequence from the neurogenic locus Notch implies a gene product that shares homology with proteins containing EGF-like repeats. Cell.

[5] Bellavia D., Checquolo S., Campese A.F., Felli M.P., Gulino A., Screpanti I. (2008). Notch3: From subtle structural differences to functional diversity. Oncogene.

[6] Hori K., Sen A., Artavanis-Tsakonas S. (2013). Notch signaling at a glance. J. Cell Sci..

[7] Gordon W.R., Vardar-Ulu D., Histen G., Sanchez-Irizarry C., Aster J.C., Blacklow S.C. (2007). Structural basis for autoinhibition of Notch. Nat. Struct. Mol. Biol..

[8] Gordon W.R., Vardar-Ulu D., L’Heureux S., Ashworth T., Malecki M.J., Sanchez-Irizarry C., McArthur D.G., Histen G., Mitchell J.L., Aster J.C. (2009). Effects of S1 cleavage on the structure, surface export, and signaling activity of human Notch1 and Notch2. PLoS ONE.

[9] Stephenson N.L., Avis J.M. (2012). Direct observation of proteolytic cleavage at the S2 site upon forced unfolding of the Notch negative regulatory region. Proc. Natl. Acad. Sci. USA.

[10] Gordon W.R., Zimmerman B., He L., Miles L.J., Huang J., Tiyanont K., McArthur D.G., Aster J.C., Perrimon N., Loparo J.J. (2015). Mechanical allostery: Evidence for a force requirement in the proteolytic activation of Notch. Dev. Cell.

[11] Struhl G., Adachi A. (2000). Requirements for presenilin-dependent cleavage of Notch and other transmembrane proteins. Mol. Cell.

[12] Wilkin M.B., Carbery A.M., Fostier M., Aslam H., Mazaleyrat S.L., Higgs J., Myat A., Evans D.A., Cornell M., Baron M. (2004). Regulation of Notch endosomal sorting and signaling by Drosophila Nedd4 family proteins. Curr. Biol..

[13] Wilkin M., Tongngok P., Gensch N., Clemence S., Motoki M., Yamada K., Hori K., Taniguchi-Kanai M., Franklin E., Matsuno K. (2008). Drosophila HOPS and AP-3 complex genes are required for a Deltex-regulated activation of notch in the endosomal trafficking pathway. Dev. Cell.

[14] Hori K., Sen A., Kirchhausen T., Artavanis-Tsakonas S. (2011). Synergy between the ESCRT-III complex and Deltex defines a ligand-independent Notch signal. J. Cell Biol..

[15] Mukherjee T., Kim W.S., Mandal L., Banerjee U. (2011). Interaction between Notch and Hif-alpha in development and survival of Drosophila blood cells. Science.

[16] Zheng L., Saunders C.A., Sorensen E.B., Waxmonsky N.C., Conner S.D. (2013). Notch signaling from the endosome requires a conserved dileucine motif. Mol. Biol. Cell.

[17] Nemetschke L., Knust E. (2016). Drosophila Crumbs prevents ectopic Notch activation in developing wings by inhibiting ligand-independent endocytosis. Development.

[18] Steinbuck M.P., Winandy S. (2018). A review of notch processing with new insights into ligand-independent notch signaling in T-Cells. Front. Immunol..

[19] Schneider M., Troost T., Grawe F., Martinez-Arias A., Klein T. (2013). Activation of Notch in lgd mutant cells requires the fusion of late endosomes with the lysosome. J. Cell Sci..

[20] Shimizu H., Woodcock S.A., Wilkin M.B., Trubenová B., Monk N.A., Baron M. (2014). Compensatory flux changes within an endocytic trafficking network maintain thermal robustness of Notch signaling. Cell.

[21] Shimizu K., Chiba S., Saito T., Kumano K., Hirai H. (2000). Physical interaction of Delta1, Jagged1, and Jagged2 with Notch1 and Notch3 receptors. Biochem. Biophys. Res. Commun..

[22] Peters N., Opherk C., Zacherle S., Capell A., Gempel P., Dichgans M. (2004). CADASIL-associated Notch3 mutations have differential effects both on ligand binding and ligand-induced Notch3 receptor signaling through RBP-Jk. Exp. Cell Res..

[23] Liu H., Kennard S., Lilly B. (2009). NOTCH3 expression is induced in mural cells through an autoregulatory loop that requires endothelial-expressed JAGGED1. Circ. Res..

[24] Xia Y., Bhattacharyya A., Roszell E.E., Sandig M., Mequanint K. (2012). The role of endothelial cell-bound Jagged1 in Notch3-induced human coronary artery smooth muscle cell differentiation. Biomaterials.

[25] Xu X., Choi S.H., Hu T., Tiyanont K., Habets R., Groot A.J., Vooijs M., Aster J.C., Chopra R., Fryer C. (2015). Insights into autoregulation of notch3 from structural and functional studies of its negative regulatory region. Structure.

[26] Rauen T., Raffetseder U., Frye B.C., Djudjaj S., Mühlenberg P.J., Eitner F., Lendahl U., Bernhagen J., Dooley S., Mertens P.R. (2009). YB-1 acts as a ligand for Notch-3 receptors and modulates receptor activation. J. Biol. Chem..

[27] Gera S., Dighe R.R. (2018). The soluble ligand Y box-1 activates Notch3 receptor by binding to epidermal growth factor like repeats 20–23. Arch. Biochem. Biophys..

[28] Krebs L.T., Xue Y., Norton C.R., Sundberg J.P., Beatus P., Lendahl U., Joutel A., Gridley T. (2003). Characterization of Notch3-deficient mice: Normal embryonic development and absence of genetic interactions with a Notch1 mutation. Genesis.

[29] Carmeliet P. (2003). Angiogenesis in health and disease. Nat. Med..

[30] Domenga V., Fardoux P., Lacombe P., Monet M., Maciazek J., Krebs L.T., Klonjkowski B., Berrou E., Mericskay M., Li Z. (2004). Notch3 is required for arterial identity and maturation of vascular smooth muscle cells. Genes Dev..

[31] Jin S., Hansson E.M., Tikka S., Lanner F., Sahlgren C., Farnebo F., Baumann M., Kalimo H., Lendahl U. (2008). Notch signaling regulates platelet-derived growth factor receptor-beta expression in vascular smooth muscle cells. Circ. Res..

[32] Ando K., Wang W., Peng D., Chiba A., Lagendijk A.K., Barske L., Crump J.G., Stainier D.Y.R., Lendahl U., Koltowska K. (2019). Peri-arterial specification of vascular mural cells from naïve mesenchyme requires Notch signaling. Development.

[33] Wang Q., Zhao N., Kennard S., Lilly B. (2012). Notch2 and Notch3 function together to regulate vascular smooth muscle development. PLoS ONE.

[34] Baeten J.T., Lilly B. (2015). Differential regulation of NOTCH2 and NOTCH3 contribute to their unique functions in vascular smooth muscle cells. J. Biol. Chem..

[35] Boucher J.M., Harrington A., Rostama B., Lindner V., Liaw L. (2013). A receptor-specific function for Notch2 in mediating vascular smooth muscle cell growth arrest through cyclin-dependent kinase inhibitor 1B. Circ. Res..

[36] Kofler N.M., Cuervo H., Uh M.K., Murtomäki A., Kitajewski J. (2015). Combined deficiency of Notch1 and Notch3 causes pericyte dysfunction, models CADASIL, and results in arteriovenous malformations. Sci. Rep..

[37] Wang Y., Pan L., Moens C.B., Appel B. (2014). Notch3 establishes brain vascular integrity by regulating pericyte number. Development.

[38] Volz K.S., Jacobs A.H., Chen H.I., Poduri A., McKay A.S., Riordan D.P., Kofler N., Kitajewski J., Weissman I., Red-Horse K. (2015). Pericytes are progenitors for coronary artery smooth muscle. Elife.

[39] Qiu X., Lim C.H., Ho S.H., Lee K.H., Jiang Y.J. (2009). Temporal Notch activation through Notch1a and Notch3 is required for maintaining zebrafish rhombomere boundaries. Dev. Genes Evol..

[40] Belin de Chantemèle E.J., Retailleau K., Pinaud F., Vessières E., Bocquet A., Guihot A.L., Lemaire B., Domenga V., Baufreton C., Loufrani L. (2008). Notch3 is a major regulator of vascular tone in cerebral and tail resistance arteries. Arterioscler. Thromb. Vasc. Biol..

[41] Frismantiene A., Philippova M., Erne P., Resink T.J. (2018). Smooth muscle cell-driven vascular diseases and molecular mechanisms of VSMC plasticity. Cell Signal..

[42] Loerakker S., Stassen O.M.J.A., Ter Huurne F.M., Boareto M., Bouten C.V.C., Sahlgren C.M. (2018). Mechanosensitivity of Jagged-Notch signaling can induce a switch-type behavior in vascular homeostasis. Proc. Natl. Acad. Sci. USA.

[43] Driessen R.C.H., Stassen O.M.J.A., Sjöqvist M., Suarez Rodriguez F., Grolleman J., Bouten C.V.C., Sahlgren C.M. (2018). Shear stress induces expression, intracellular reorganization and enhanced Notch activation potential of Jagged1. Integr. Biol..

[44] Kawai H., Kawaguchi D., Kuebrich B.D., Kitamoto T., Yamaguchi M., Gotoh Y., Furutachi S. (2017). Area-Specific Regulation of Quiescent Neural Stem Cells by Notch3 in the Adult Mouse Subependymal Zone. J. Neurosci..

[45] Alunni A., Krecsmarik M., Bosco A., Galant S., Pan L., Moens C.B., Bally-Cuif L. (2013). Notch3 signaling gates cell cycle entry and limits neural stem cell amplification in the adult pallium. Development.

[46] Kitamoto T., Hanaoka K. (2010). Notch3 null mutation in mice causes muscle hyperplasia by repetitive muscle regeneration. Stem Cells.

[47] Conboy I.M., Rando T.A. (2002). The regulation of Notch signaling controls satellite cell activation and cell fate determination in postnatal myogenesis. Dev. Cell.

[48] Lafkas D., Rodilla V., Huyghe M., Mourao L., Kiaris H., Fre S. (2013). Notch3 marks clonogenic mammary luminal progenitor cells in vivo. J. Cell Biol..

[49] Ohashi S., Natsuizaka M., Yashiro-Ohtani Y., Kalman R.A., Nakagawa M., Wu L., Klein-Szanto A.J., Herlyn M.V., Diehl J.A., Katz J.P. (2010). NOTCH1 and NOTCH3 coordinate esophageal squamous differentiation through a CSL-dependent transcriptional network. Gastroenterology.

[50] Ellisen L.W., Bird J., West D.C., Soreng A.L., Reynolds T.C., Smith S.D., Sklar J. (1991). TAN-1, the human homolog of the Drosophila Notch gene, is broken by chromosomal translocations in T lymphoblastic neoplasms. Cell.

[51] Weng A.P., Ferrando A.A., Lee W., Morris J.P., Silverman L.B., Sanchez-Irizarry C., Blacklow S.C., Look A.T., Aster J.C. (2004). Activating mutations of NOTCH1 in human T cell acute lymphoblastic leukemia. Science.

[52] Bernasconi-Elias P., Hu T., Jenkins D., Firestone B., Gans S., Kurth E., Capodieci P., Deplazes-Lauber J., Petropoulos K., Thiel P. (2016). Characterization of activating mutations of NOTCH3 in T-cell acute lymphoblastic leukemia and anti-leukemic activity of NOTCH3 inhibitory antibodies. Oncogene.

[53] Bellavia D., Campese A.F., Checquolo S., Balestri A., Biondi A., Cazzaniga G., Lendahl U., Fehling H.J., Hayday A.C., Frati L. (2002). Combined expression of pTalpha and Notch3 in T cell leukemia identifies the requirement of preTCR for leukemogenesis. Proc. Natl. Acad. Sci. USA.

[54] Franciosa G., Diluvio G., Gaudio F.D., Giuli M.V., Palermo R., Grazioli P., Campese A.F., Talora C., Bellavia D., D’Amati G.V. (2016). Prolyl-isomerase Pin1 controls Notch3 protein expression and regulates T-ALL progression. Oncogene.

[55] Choi S.H., Severson E., Pear W.S., Liu X.S., Aster J.C., Blacklow S.C. (2017). The common oncogenomic program of NOTCH1 and NOTCH3 signaling in T-cell acute lymphoblastic leukemia. PLoS ONE.

[56] Wang H., Zang C., Taing L., Arnett K.L., Wong Y.J., Pear W.S., Blacklow S.C., Liu X.S., Aster J.C. (2014). NOTCH1-RBPJ complexes drive target gene expression through dynamic interactions with superenhancers. Proc. Natl. Acad. Sci. USA.

[57] Tottone L., Zhdanovskaya N., Carmona Pestaña Á, Zampieri M., Simeoni F., Lazzari S., Ruocco V., Pelullo M., Caiafa P., Felli M.P. (2019). Histone Modifications Drive Aberrant Notch3 Expression/Activity and Growth in T-ALL. Front. Oncol..

[58] Zampieri M., Ciccarone F., Palermo R., Cialfi S., Passananti C., Chiaretti S., Nocchia D., Talora C., Screpanti I., Caiafa P. (2014). The epigenetic factor BORIS/CTCFL regulates the NOTCH3 gene expression in cancer cells. Biochim. Biophys. Acta.

[59] Ghisi M., Corradin A., Basso K., Frasson C., Serafin V., Mukherjee S., Mussolin L., Ruggero K., Bonanno L., Guffanti A. (2011). Modulation of microRNA expression in human T-cell development: Targeting of NOTCH3 by miR-150. Blood.

[60] Podshivalova K., Wang E.A., Hart T., Salomon D.R. (2018). Expression of the miR-150 tumor suppressor is restored by and synergizes with rapamycin in a human leukemia T-cell line. Leuk. Res..

[61] Zhang Y., Chen B., Wang Y., Zhao Q., Wu W., Zhang P., Miao L., Sun S. (2019). NOTCH3 Overexpression and Posttranscriptional Regulation by miR-150 Were Associated With EGFR-TKI Resistance in Lung Adenocarcinoma. Oncol. Res..

[62] Kim T.H., Jeong J.Y., Park J.Y., Kim S.W., Heo J.H., Kang H., Kim G., An H.J. (2017). miR-150 enhances apoptotic and anti-tumor effects of paclitaxel in paclitaxel-resistant ovarian cancer cells by targeting Notch3. Oncotarget.

[63] Pinazza M., Ghisi M., Minuzzo S., Agnusdei V., Fossati G., Ciminale V., Pezzè L., Ciribilli Y., Pilotto G., Venturoli C. (2018). Histone deacetylase 6 controls Notch3 trafficking and degradation in T-cell acute lymphoblastic leukemia cells. Oncogene.

[64] Palermo R., Checquolo S., Giovenco A., Grazioli P., Kumar V., Campese A.F., Giorgi A., Napolitano M., Canettieri G., Ferrara G. (2012). Acetylation controls Notch3 stability and function in T-cell leukemia. Oncogene.

[65] Indraccolo S., Minuzzo S., Masiero M., Pusceddu I., Persano L., Moserle L., Reboldi A., Favaro E., Mecarozzi M., Di Mario G. (2009). Cross-talk between tumor and endothelial cells involving the Notch3-Dll4 interaction marks escape from tumor dormancy. Cancer Res..

[66] Minuzzo S., Agnusdei V., Pusceddu I., Pinazza M., Moserle L., Masiero M., Rossi E., Crescenzi M., Hoey T., Ponzoni M. (2015). DLL4 regulates NOTCH signaling and growth of T acute lymphoblastic leukemia cells in NOD/SCID mice. Carcinogenesis.

[67] Pelullo M., Quaranta R., Talora C., Checquolo S., Cialfi S., Felli M.P., te Kronnie G., Borga C., Besharat Z.M., Palermo R. (2014). Notch3/Jagged1 circuitry reinforces Notch signaling and sustains T-ALL. Neoplasia.

[68] Choy L., Hagenbeek T.J., Solon M., French D., Finkle D., Shelton A., Venook R., Brauer M.J., Siebel C.W. (2017). Constitutive NOTCH3 Signaling Promotes the Growth of Basal Breast Cancers. Cancer Res..

[69] Leontovich A.A., Jalalirad M., Salisbury J.L., Mills L., Haddox C., Schroeder M., Tuma A., Guicciardi M.E., Zammataro L., Gambino M.W. (2018). NOTCH3 expression is linked to breast cancer seeding and distant metastasis. Breast Cancer Res..

[70] Wang K., Zhang Q., Li D., Ching K., Zhang C., Zheng X., Ozeck M., Shi S., Li X., Wang H. (2015). PEST domain mutations in Notch receptors comprise an oncogenic driver segment in triple-negative breast cancer sensitive to a γ-secretase inhibitor. Clin. Cancer Res..

[71] Jung J.G., Stoeck A., Guan B., Wu R.C., Zhu H., Blackshaw S., Shih I.E.M., Wang T.L. (2014). Notch3 interactome analysis identified WWP2 as a negative regulator of Notch3 signaling in ovarian cancer. PLoS Genet..

[72] Lin H.Y., Liang Y.K., Dou X.W., Chen C.F., Wei X.L., Zeng D., Bai J.W., Guo Y.X., Lin F.F., Huang W.H. (2018). Notch3 inhibits epithelial-mesenchymal transition in breast cancer via a novel mechanism, upregulation of GATA-3 expression. Oncogenesis.

[73] Sourander P., Wålinder J. (1977). Hereditary multi-infarct dementia. Morphological and clinical studies of a new disease. Acta Neuropathol..

[74] Joutel A., Vahedi K., Corpechot C., Troesch A., Chabriat H., Vayssière C., Cruaud C., Maciazek J., Weissenbach J., Bousser M.G. (1997). Strong clustering and stereotyped nature of Notch3 mutations in CADASIL patients. Lancet.

[75] Dichgans M., Mayer M., Uttner I., Brüning R., Müller-Höcker J., Rungger G., Ebke M., Klockgether T., Gasser T. (1998). The phenotypic spectrum of CADASIL: Clinical findings in 102 cases. Ann. Neurol..

[76] Louvi A., Arboleda-Velasquez J.F., Artavanis-Tsakonas S. (2006). CADASIL: A critical look at a Notch disease. Dev. Neurosci..

[77] Joutel A., Andreux F., Gaulis S., Domenga V., Cecillon M., Battail N., Piga N., Chapon F., Godfrain C., Tournier-Lasserve E. (2000). The ectodomain of the Notch3 receptor accumulates within the cerebrovasculature of CADASIL patients. J. Clin. Invest..

[78] Joutel A., Favrole P., Labauge P., Chabriat H., Lescoat C., Andreux F., Domenga V., Cécillon M., Vahedi K., Ducros A. (2001). Skin biopsy immunostaining with a Notch3 monoclonal antibody for CADASIL diagnosis. Lancet.

[79] Monet-Leprêtre M., Haddad I., Baron-Menguy C., Fouillot-Panchal M., Riani M., Domenga-Denier V., Dussaule C., Cognat E., Vinh J., Joutel A. (2013). Abnormal recruitment of extracellular matrix proteins by excess Notch3 ECD: A new pathomechanism in CADASIL. Brain.

[80] Yamamoto Y., Craggs L.J., Watanabe A., Booth T., Attems J., Low R.W., Oakley A.E., Kalaria R.N. (2013). Brain microvascular accumulation and distribution of the NOTCH3 ectodomain and granular osmiophilic material in CADASIL. J. Neuropathol. Exp. Neurol..

[81] Gray F., Polivka M., Viswanathan A., Baudrimont M., Bousser M.G., Chabriat H. (2007). Apoptosis in cerebral autosomal-dominant arteriopathy with subcortical infarcts and leukoencephalopathy. J. Neuropathol. Exp. Neurol..

[82] Panahi M., Yousefi Mesri N., Samuelsson E.B., Coupland K.G., Forsell C., Graff C., Tikka S., Winblad B., Viitanen M., Karlström H. (2018). Differences in proliferation rate between CADASIL and control vascular smooth muscle cells are related to increased TGFβ expression. J. Cell Mol. Med..

[83] Rajani R.M., Ratelade J., Domenga-Denier V., Hase Y., Kalimo H., Kalaria R.N., Joutel A. (2019). Blood brain barrier leakage is not a consistent feature of white matter lesions in CADASIL. Acta Neuropathol. Commun..

[84] Rutten J.W., Dauwerse H.G., Gravesteijn G., van Belzen M.J., van der Grond J., Polke J.M., Bernal-Quiros M., Lesnik Oberstein S.A. (2016). Archetypal NOTCH3 mutations frequent in public exome: Implications for CADASIL. Ann. Clin. Transl. Neurol..

[85] Rutten J.W., Van Eijsden B.J., Duering M., Jouvent E., Opherk C., Pantoni L., Federico A., Dichgans M., Markus H.S., Chabriat H. (2019). The effect of NOTCH3 pathogenic variant position on CADASIL disease severity: NOTCH3 EGFr 1-6 pathogenic variant are associated with a more severe phenotype and lower survival compared with EGFr 7-34 pathogenic variant. Genet. Med..

[86] Kelley M.R., Kidd S., Deutsch W.A., Young M.W. (1987). Mutations altering the structure of epidermal growth factor-like coding sequences at the Drosophila Notch locus. Cell.

[87] Bardot B., Mok L.P., Thayer T., Ahimou F., Wesley C. (2005). The Notch amino terminus regulates protein levels and Delta-induced clustering of Drosophila Notch receptors. Exp. Cell Res..

[88] Arboleda-Velasquez J.F., Lopera F., Lopez E., Frosch M.P., Sepulveda-Falla D., Gutierrez J.E., Vargas S., Medina M., Martinez De Arrieta C., Lebo R.V. (2002). C455R Notch3 mutation in a Colombian CADASIL kindred with early onset of stroke. Neurology.

[89] Monet-Leprêtre M., Bardot B., Lemaire B., Domenga V., Godin O., Dichgans M., Tournier-Lasserve E., Cohen-Tannoudji M., Chabriat H., Joutel A. (2009). Distinct phenotypic and functional features of CADASIL mutations in the Notch3 ligand binding domain. Brain.

[90] Joutel A., Monet M., Domenga V., Riant F., Tournier-Lasserve E. (2004). Pathogenic mutations associated with cerebral autosomal dominant arteriopathy with subcortical infarcts and leukoencephalopathy differently affect Jagged1 binding and Notch3 activity via the RBP/JK signaling Pathway. Am. J. Hum. Genet..

[91] Monet M., Domenga V., Lemaire B., Souilhol C., Langa F., Babinet C., Gridley T., Tournier-Lasserve E., Cohen-Tannoudji M., Joutel A. (2007). The archetypal R90C CADASIL-NOTCH3 mutation retains NOTCH3 function in vivo. Hum. Mol. Genet..

[92] Kast J., Hanecker P., Beaufort N., Giese A., Joutel A., Dichgans M., Opherk C., Haffner C. (2014). Sequestration of latent TGF-β binding protein 1 into CADASIL-related Notch3-ECD deposits. Acta Neuropathol. Commun..

[93] Capone C., Cognat E., Ghezali L., Baron-Menguy C., Aubin D., Mesnard L., Stöhr H., Domenga-Denier V., Nelson M.T., Joutel A. (2016). Reducing Timp3 or vitronectin ameliorates disease manifestations in CADASIL mice. Ann. Neurol..

[94] Dotti M.T., Federico A., Mazzei R., Bianchi S., Scali O., Conforti F.L., Sprovieri T., Guidetti D., Aguglia U., Consoli D. (2005). The spectrum of Notch3 mutations in 28 Italian CADASIL families. J. Neurol. Neurosurg. Psychiatry.

[95] Moccia M., Mosca L., Erro R., Cervasio M., Allocca R., Vitale C., Leonardi A., Caranci F., Del Basso-De Caro M.L., Barone P. (2015). Hypomorphic NOTCH3 mutation in an Italian family with CADASIL features. Neurobiol. Aging.

[96] Rutten J.W., Boon E.M., Liem M.K., Dauwerse J.G., Pont M.J., Vollebregt E., Maat-Kievit A.J., Ginjaar H.B., Lakeman P., van Duinen S.G. (2013). Hypomorphic NOTCH3 alleles do not cause CADASIL in humans. Hum. Mutat..

[97] Lesnik Oberstein S.A., van Duinen S.G., van den Boom R., Maat-Schieman M.L., van Buchem M.A., van Houwelingen H.C., Hegeman-Kleinn I.M., Ferrari M.D., Breuning M.H., Haan J. (2003). Evaluation of diagnostic NOTCH3 immunostaining in CADASIL. Acta Neuropathol..

[98] Pippucci T., Maresca A., Magini P., Cenacchi G., Donadio V., Palombo F., Papa V., Incensi A., Gasparre G., Valentino M.L. (2015). Homozygous NOTCH3 null mutation and impaired NOTCH3 signaling in recessive early-onset arteriopathy and cavitating leukoencephalopathy. EMBO Mol. Med..

[99] Henshall T.L., Keller A., He L., Johansson B.R., Wallgard E., Raschperger E., Mäe M.A., Jin S., Betsholtz C., Lendahl U. (2015). Notch3 is necessary for blood vessel integrity in the central nervous system. Arterioscler. Thromb. Vasc. Biol..

[100] Cognat E., Baron-Menguy C., Domenga-Denier V., Cleophax S., Fouillade C., Monet-Leprêtre M., Dewerchin M., Joutel A. (2014). Archetypal Arg169Cys mutation in NOTCH3 does not drive the pathogenesis in cerebral autosomal dominant arteriopathy with subcortical infarcts and leucoencephalopathy via a loss-of-function mechanism. Stroke.

[101] Meng H., Zhang X., Yu G., Lee S.J., Chen Y.E., Prudovsky I., Wang M.M. (2012). Biochemical characterization and cellular effects of CADASIL mutants of NOTCH3. PLoS ONE.

[102] Arboleda-Velasquez J.F., Manent J., Lee J.H., Tikka S., Ospina C., Vanderburg C.R., Frosch M.P., Rodríguez-Falcón M., Villen J., Gygi S. (2011). Hypomorphic Notch 3 alleles link Notch signaling to ischemic cerebral small-vessel disease. Proc. Natl. Acad. Sci. USA.

[103] Machuca-Parra A.I., Bigger-Allen A.A., Sanchez A.V., Boutabla A., Cardona-Vélez J., Amarnani D., Saint-Geniez M., Siebel C.W., Kim L.A., D’Amore P.A. (2017). Therapeutic antibody targeting of Notch3 signaling prevents mural cell loss in CADASIL. J. Exp. Med..

[104] Ghezali L., Capone C., Baron-Menguy C., Ratelade J., Christensen S., Østergaard Pedersen L., Domenga-Denier V., Pedersen J.T., Joutel A. (2018). Notch3 (ECD) immunotherapy improves cerebrovascular responses in CADASIL mice. Ann. Neurol..

[105] Neves K.B., Harvey A.P., Moreton F., Montezano A.C., Rios F.J., Alves-Lopes R., Nguyen Dinh Cat A., Rocchicciolli P., Delles C., Joutel A. (2019). ER stress and Rho kinase activation underlie the vasculopathy of CADASIL. JCI Insight.

[106] Ling C., Liu Z., Song M., Zhang W., Wang S., Liu X., Ma S., Sun S., Fu L., Chu Q. (2019). Modeling CADASIL vascular pathologies with patient-derived induced pluripotent stem cells. Protein Cell.

[107] Kelleher J., Dickinson A., Cain S., Hu Y., Bates N., Harvey A., Ren J., Zhang W., Moreton F.C., Muir K.W. (2019). Patient-Specific iPSC Model of a Genetic Vascular Dementia Syndrome Reveals Failure of Mural Cells to Stabilize Capillary Structures. Stem Cell Rep..

[108] Rand M.D., Grimm L.M., Artavanis-Tsakonas S., Patriub V., Blacklow S.C., Sklar J., Aster J.C. (2000). Calcium depletion dissociates and activates heterodimeric notch receptors. Mol. Cell Biol..

[109] Takahashi K., Adachi K., Yoshizaki K., Kunimoto S., Kalaria R.N., Watanabe A. (2010). Mutations in NOTCH3 cause the formation and retention of aggregates in the endoplasmic reticulum, leading to impaired cell proliferation. Hum. Mol. Genet..

[110] Watanabe-Hosomi A., Watanabe Y., Tanaka M., Nakagawa M., Mizuno T. (2012). Transendocytosis is impaired in CADASIL-mutant NOTCH3. Exp. Neurol..

[111] Dziewulska D., Rafalowska J. (2008). Is the increased expression of ubiquitin in CADASIL syndrome a manifestation of aberrant endocytosis in the vascular smooth muscle cells?. J. Clin. Neurosci..

[112] Hanemaaijer E.S., Panahi M., Swaddiwudhipong N., Tikka S., Winblad B., Viitanen M., Piras A., Behbahani H. (2018). Autophagy-lysosomal defect in human CADASIL vascular smooth muscle cells. Eur. J. Cell Biol..

[113] Gravesteijn G., Dauwerse J.G., Overzier M., Brouwer G., Hegeman I., Mulder A.A., Baas F., Kruit M.C., Terwindt G.M., van Duinen S.G. (2020). Naturally occurring NOTCH3 exon skipping attenuates NOTCH3 protein aggregation and disease severity in CADASIL patients. Hum. Mol. Genet..

[114] Li X., Zhang X., Leathers R., Makino A., Huang C., Parsa P., Macias J., Yuan J.X., Jamieson S.W., Thistlethwaite P.A. (2009). Notch3 signaling promotes the development of pulmonary arterial hypertension. Nat. Med..

[115] Wang W., Liu J., Ma A., Miao R., Jin Y., Zhang H., Xu K., Wang C., Wang J. (2014). mTORC1 is involved in hypoxia-induced pulmonary hypertension through the activation of Notch3. J. Cell Physiol..

[116] Wang Y., Dai S., Cheng X., Prado E., Yan L., Hu J., He Q., Lv Y., Lv Y., Du L. (2019). Notch3 signaling activation in smooth muscle cells promotes extrauterine growth restriction-induced pulmonary hypertension. Nutr. Metab. Cardiovasc. Dis..

[117] Chida A., Shintani M., Matsushita Y., Sato H., Eitoku T., Nakayama T., Furutani Y., Hayama E., Kawamura Y., Inai K. (2014). Mutations of NOTCH3 in childhood pulmonary arterial hypertension. Mol. Genet. Genomic Med..

